# Glycol chitosan-based tacrolimus-loaded nanomicelle therapy ameliorates lupus nephritis

**DOI:** 10.1186/s12951-021-00857-w

**Published:** 2021-04-17

**Authors:** Chang Seong Kim, Ansuja Pulickal Mathew, Arathy Vasukutty, Saji Uthaman, Soo Yeon Joo, Eun Hui Bae, Seong Kwon Ma, In-Kyu Park, Soo Wan Kim

**Affiliations:** 1 Department of Internal Medicine, Chonnam National University Medical School, 160, Baekseo-ro, Dong-gu, Gwangju, 61496 Republic of Korea; 2 Department of Internal Medicine, Chonnam National University Hospital, Gwangju, Republic of Korea; 3 Department of Biomedical Sciences, BioMedical Sciences Graduate Program (BMSGP), Chonnam National University, Hwasun, 58128 Republic of Korea; 4 Department of Polymer Science and Engineering, Chungnam National University, Daejeon, 34134 Republic of Korea

**Keywords:** Nanomicelles, Chitosan, Adherence, Tacrolimus, Lupus nephritis, Kidney injury

## Abstract

**Background:**

Recently, we developed hydrophobically modified glycol chitosan (HGC) nanomicelles loaded with tacrolimus (TAC) (HGC-TAC) for the targeted renal delivery of TAC. Herein, we determined whether the administration of the HGC-TAC nanomicelles decreases kidney injury in a model of lupus nephritis. Lupus-prone female MRL/lpr mice were randomly assigned into three groups that received intravenous administration of either vehicle control, an equivalent dose of TAC, or HGC-TAC (0.5 mg/kg TAC) weekly for 8 weeks. Age-matched MRL/MpJ mice without *Fas*^*lpr*^ mutation were also treated with HGC vehicle and used as healthy controls.

**Results:**

Weekly intravenous treatment with HGC-TAC significantly reduced genetically attributable lupus activity in lupus nephritis-positive mice. In addition, HGC-TAC treatment mitigated renal dysfunction, proteinuria, and histological injury, including glomerular proliferative lesions and tubulointerstitial infiltration. Furthermore, HGC-TAC treatment reduced renal inflammation and inflammatory gene expression and ameliorated increased apoptosis and glomerular fibrosis. Moreover, HGC-TAC administration regulated renal injury via the TGF-β1/MAPK/NF-κB signaling pathway. These renoprotective effects of HGC-TAC treatment were more potent in lupus mice compared to those of TAC treatment alone.

**Conclusion:**

Our study indicates that weekly treatment with the HGC-TAC nanomicelles reduces kidney injury resulting from lupus nephritis by preventing inflammation, fibrosis, and apoptosis. This advantage of a new therapeutic modality using kidney-targeted HGC-TAC nanocarriers may improve drug adherence and provide treatment efficacy in lupus nephritis mice.

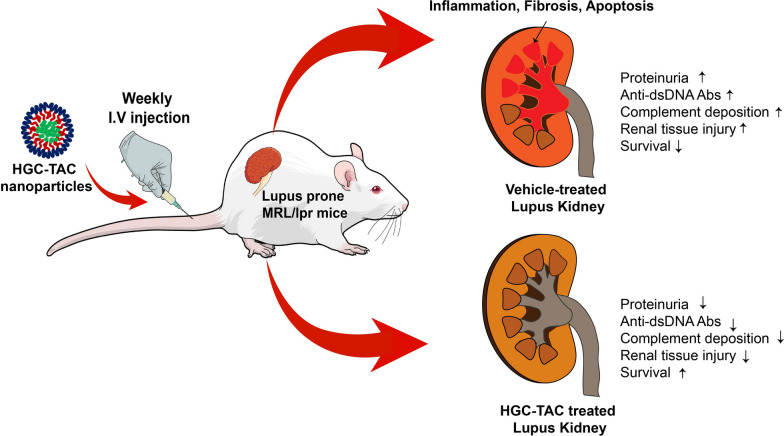

**Supplementary Information:**

The online version contains supplementary material available at 10.1186/s12951-021-00857-w.

## Background

Systemic lupus erythematosus (SLE) is an autoimmune disease characterized by the production of autoantibodies against cell nuclear components that can affect any organ, including the kidneys [1]. Varying degrees of renal involvement (ranging from 30 to 60% and dependent upon both ethnicity and lesion type) are seen in patients with SLE [2–4]. Lupus nephritis may progress into end-stage kidney disease and is independently associated with higher morbidity and mortality, even among patients undergoing dialysis and those who have undergone transplantation [2]. Therefore, patients with lupus nephritis require appropriate and continuous immunosuppressive treatment to mitigate lupus and improve kidney outcomes. Although induction therapy using standard or low-dose cyclophosphamide is important to attenuate intrarenal inflammation immediately, long-term use of a calcineurin inhibitor or antimetabolite to maintain autoimmunity and suppress inflammation for the prevention of flare is needed in patients with lupus nephritis [1, 5].

In a lupus mouse model, tacrolimus (TAC) monotherapy or in combination with mycophenolate mofetil (MMF) and prednisone significantly diminished proteinuria and glomerular injury by preserving synaptopodin via the reciprocal regulation of RhoA and Rac1 [6, 7]. Moreover, recent clinical studies have shown that TAC is more effective in inducing complete remission and reducing proteinuria than cyclophosphamide in patients with moderate to severe lupus nephritis [6, 8, 9]. Following several randomized studies evaluating the efficacy and safety of TAC as a maintenance treatment for lupus nephritis, TAC was approved for lupus nephritis treatment in Korea, Japan, and other Asian countries [10–12]. Due to the role of TAC as a potential therapeutic immunosuppressive agent, its use in induction and maintenance therapy for lupus nephritis has attracted considerable attention [13, 14].

However, clinical management with TAC therapy remains challenging due to its narrow therapeutic range and off-target effects on other organs, as well as the negative effect of long-term TAC use, including neurotoxicity, new-onset diabetes, and nephrotoxicity [15, 16]. In addition, the twice-daily oral administration decreased patient adherence to TAC therapy [17]. Although new extended-release TAC formulations exist, TAC needs to be administered daily, and the trough level for maintaining optimal therapeutic targeting should be checked [18]. Under this unmet need, nanomaterials incorporating therapeutic drugs can be engineered for slow release that allows a single-dose administration to achieve proper therapeutic targets [19]. Chitosan is one of the most functional biopolymers widely used as a pharmaceutical carrier for drug delivery [20]. Glycol chitosan possesses reactive amine groups that are accountable for the kidney-specific accumulation via megalin receptors present on the kidney [20–23]. Recently, we developed hydrophobically modified glycol chitosan (HGC) nanomicelles loaded with TAC (HGC-TAC) for the enhanced renal delivery of this immunosuppressive agent [23]. HGC-TAC nanomicelles delivered TAC preferentially to the kidney while lowering the plasma concentrations without any off-target effects [23].

There are currently limited experimental studies exploring the use of nanomaterials to treat glomerular diseases, including lupus nephritis [24–27]. Herein, we conducted a study to determine whether the administration of HGC-TAC nanomicelles decreased kidney injury in an MRL/lpr mouse model of lupus nephritis.

## Results

### Characterization of HGC-TAC nanomicelle

**Fig. 1:**
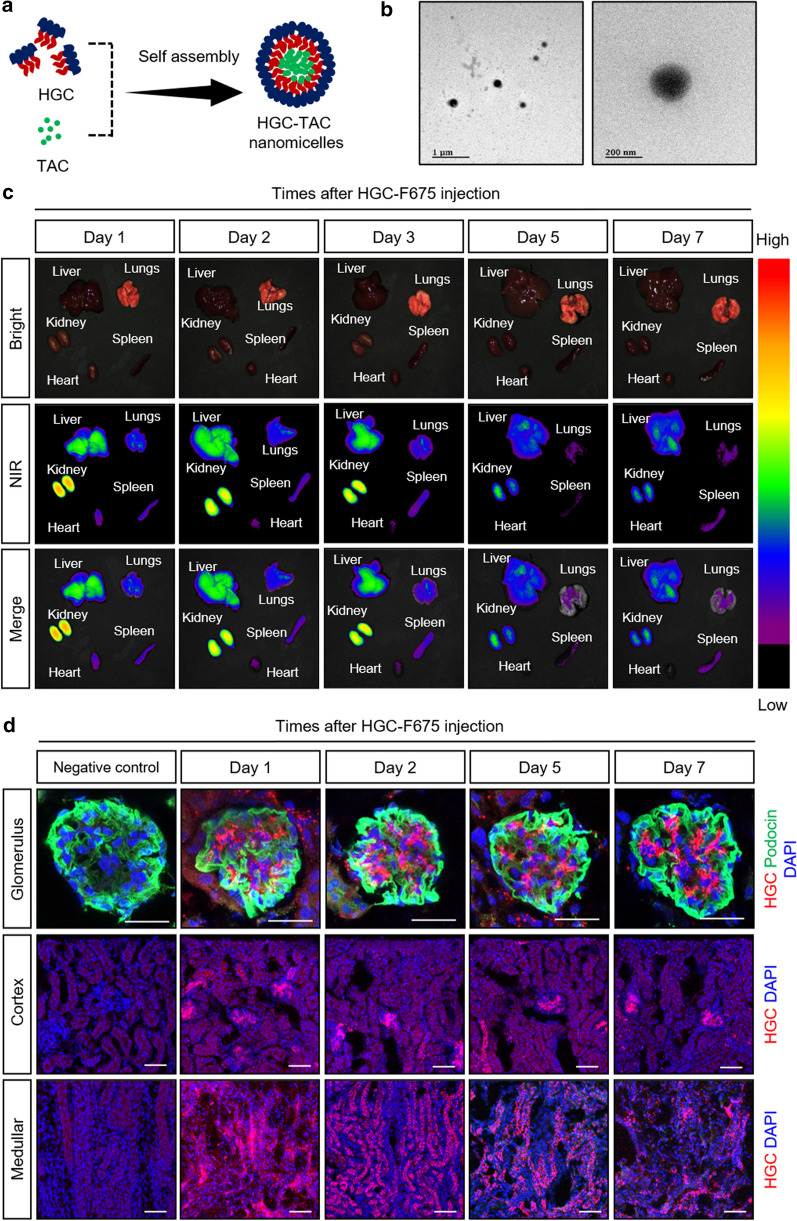
In vivo biodistribution of hydrophobically modified glycol chitosan (HGC) nanomicelles. **a** Schematic representation of the preparation of HGC nanomicelles loaded with tacrolimus (HGC-TAC). **b** The FE-TEM image of HGC-TAC nanomicelles. **c** Fluorescence images of organs at different time intervals after injection of HGC-F675 in MRL/lpr mice. The near-infrared images of dissected organs were obtained using a near-infrared filter in a fluorescence-labeled organism bio-imaging instrument. The fluorescence intensity of organs was quantified at each time point (n = 3 mice/group). **d** Confocal images of HGC-F675 nanomicelles in the kidney at different time points after injection in MRL/lpr mice. Note that podocin is a marker of podocytes, indicating the glomerulus. Original magnification ×400 or ×200, respectively. Bar = 50 μm

The hydrophobic drug, TAC, was physically encapsulated into the nanomicelles by probe sonication and dialysis (Fig. 1a). The field-emission transmission electron microscopy (FE-TEM) images of HGC-TAC nanomicelle revealed spherical morphology (Fig. 1b). The TAC loading content and encapsulating efficiency of the nanomicelle were 23 ± 3%, 88 ± 8%, respectively. The average hydrodynamic size of the HGC-TAC nanomicelle was 370 ± 22 nm per dynamic light scattering measurements. The HGC-TAC nanomicelle showed an average zeta potential of 24 ± 4 mV (Additional file 1: Fig. S1a, b). The colloidal stability of HGC-TAC nanomicelles was assessed by the time-dependent changes of the HGC-TAC nanomicelles in distilled water, PBS and, 10% FBS (Additional file 1: Fig. S1c). It was shown that in the presence of FBS, the hydrodynamic size was increased, but the zeta potential decreased over time because of the formation of protein corona over the nanomicelles. However, the polydispersity index of the particle decreased, suggesting that the particles were not destabilized. It can be assumed that the formation of protein corona prevented the aggregation of particles. To determine the time-dependent cellular uptake of HGC nanomicelles in vitro, human tubular epithelial cells were treated with Flamma 675-conjugated HGC (HGC-F675) nanomicelles. As shown in Additional file 1: Fig. S1d, fluorescence intensities increased in the cell membrane in a time-dependent manner.

### In vitro and in vivo release profile of TAC from HGC-TAC nanomicelles

The in vitro release profile of TAC in phosphate-buffered saline (PBS) and fetal bovine serum (FBS) showed biphasic and sustained release from HGC-TAC for up to 8 days (Additional file 2: Fig. S2a, b). In intravenously HGC-TAC-injected lupus mice, the plasma concentration of TAC showed a high profile at the initial hour and rapidly decreased to zero after 24 h. However, there were significant TAC concentrations in kidney tissues until at least 96 h. Therefore, encapsulated TAC in HGC nanomicelles might prevent direct TAC exposure to the plasma, keeping plasma TAC concentration low while supplying a long-lasting TAC concentration in the kidney (Additional file 2: Fig. S2c).

### In vivo biodistribution of nanomicelles

To determine the in vivo biodistribution of intravenously injected HGC nanomicelles, the fluorescence signals from various organs were serially measured for up to 7 days. As shown in Fig. 1c, the fluorescence intensity from the kidneys was the most intense compared to those from other organs after injection of HGC-F675 nanomicelles. The signal intensity from the kidneys declined gradually but was relatively well preserved for up to 7 days in MRL/lpr mice. To further localize the intrarenal distribution of the HGC-TAC nanomicelles, kidney sections were examined by confocal microscopy (Fig. 1d). HGC-F675 nanomicelle signals were localized in the cortex, medullar, and glomerular regions, suggesting a possible kidney-specific uptake of HGC-TAC nanomicelles for up to 7 days after the injection.

### HGC-TAC nanomicelle treatment attenuated lupus activity and proteinuria in lupus nephritis mice

**Fig. 2:**
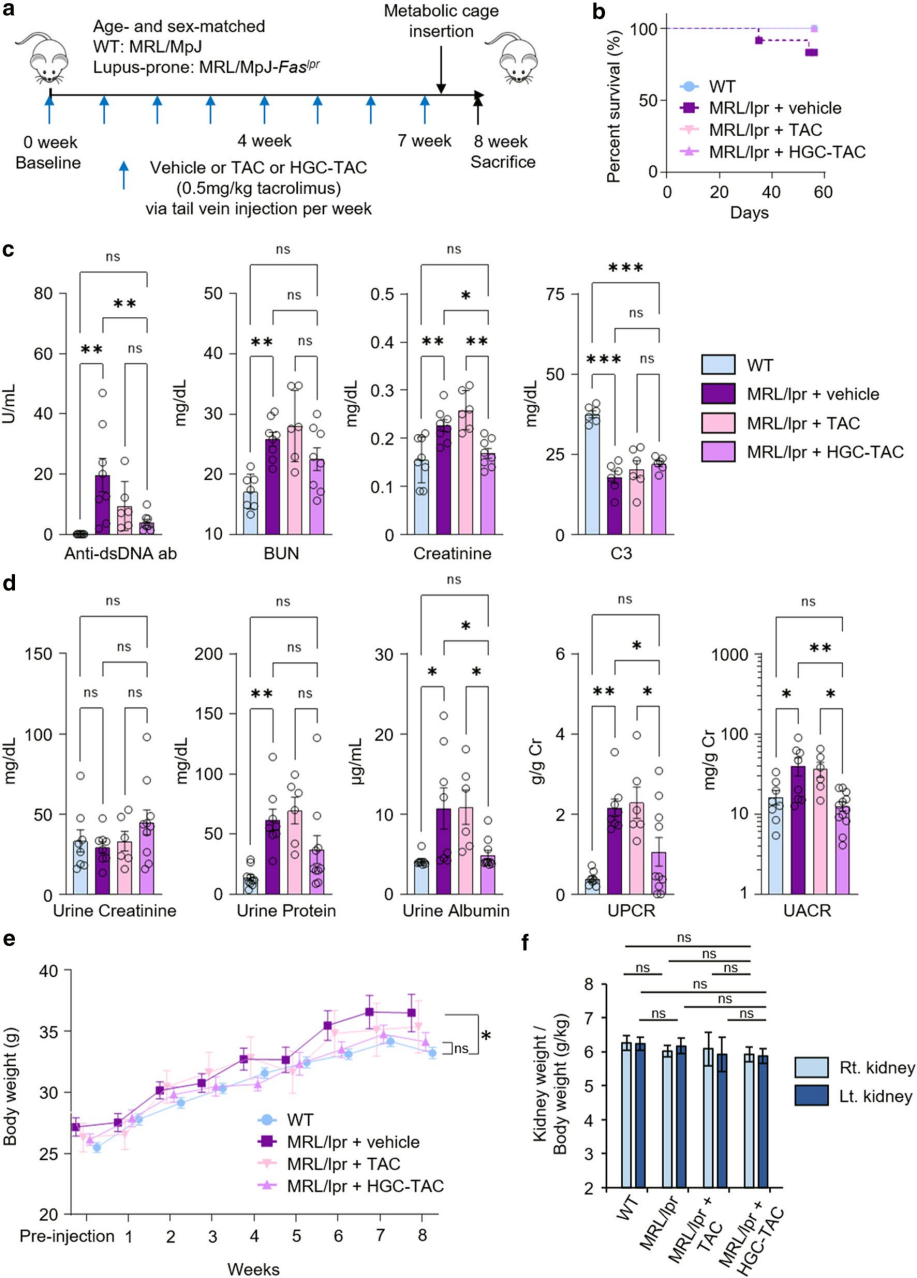
Treatment with HGC-TAC ameliorated lupus activity and proteinuria in MRL/lpr mice. **a** A schematic of the timeline of HGC-TAC treatment and experimental analysis. **b** Survival curve of the mice in each group. *WT* wild-type. **c** Serum samples were analyzed for anti-double strand DNA (anti-dsDNA) antibody in serum, serum blood urea nitrogen (BUN), serum creatinine, and complement C3 levels. **d** Protein and albumin excretion were measured in urine samples collected in metabolic cages for 24 h. Urine protein and albumin were normalized for urine creatinine. *UPCR* Urine protein-to-creatinine ratio, *UACR* urine albumin-to-creatinine ratio. **e**, **f** The body weights and the kidney-to-body weight ratio of the groups. Data are representative of three independent experiments. All values are presented as mean ± SEM. The experiment was performed with 6 mice per group. *P < 0.05, **P < 0.01, and ***P < 0.001, ^#^*P* < 0.05 HGC-TAC-treated MRL/lpr mice compared with vehicle-treated MRL/lpr mice. *ns* not statistically significant

We first investigated the effects of HGC-TAC nanomicelles on lupus activity and renal outcomes in lupus-prone MRL/lpr mice. Increased survival rates were shown in lupus mice that received HGC-TAC treatment compared to vehicle treatment (Fig. 2b). After 8 weeks, lupus mice exhibited increased anti-double-stranded DNA antibody titers and serum levels of blood urea nitrogen and creatinine and decreased serum C3 levels compared to the MRL/MpJ wild-type mice (Fig. 2c). Weekly treatment with intravenous HGC-TAC improved all these parameters, albeit serum blood urea nitrogen and C3 levels were not significantly different between vehicle- or TAC-treated lupus mice and HGC-TAC-treated lupus mice. Furthermore, HGC-TAC treatment decreased urine protein and albumin-to-creatinine ratios compared to vehicle or TAC treatment alone (Fig. 2d). Thus, HGC-TAC treatment may attenuate proteinuria and lupus nephritis activity. Although the body weights of lupus mice increased compared to wild-type mice at 8 weeks, the body weights of HGC-TAC treated lupus mice were not different from those of wild-type mice at the end of the experiment. It may be hypothesized that body edema was improved (Fig. 2e). However, there were no significant differences in kidney-to-body weight ratios among all groups (Fig. 2f).

### HGC-TAC nanomicelle treatment resulted in improved renal histology and decreased glomerular immune complex deposition in lupus nephritis mice

**Fig. 3:**
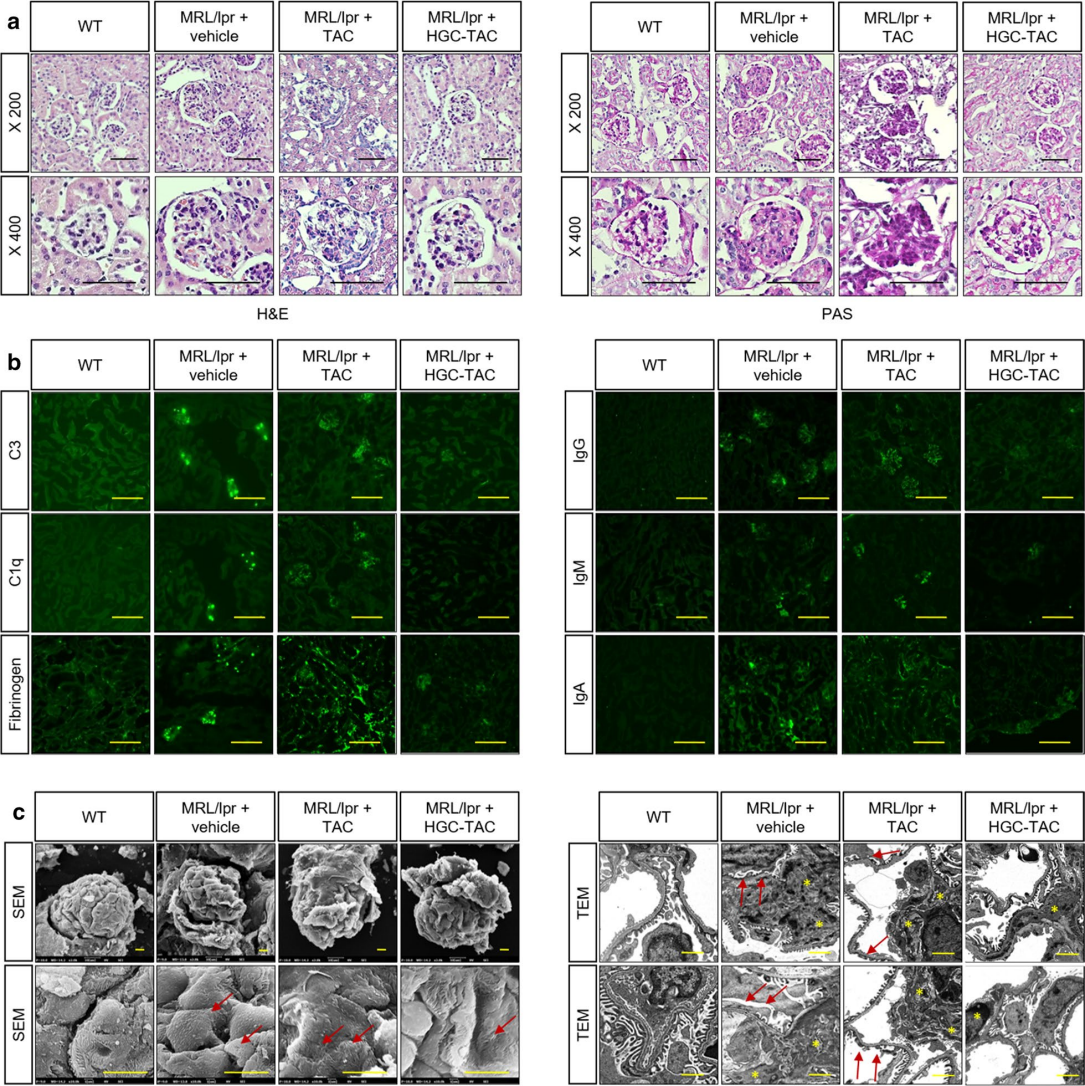
Treatment with HGC-TAC reduced glomerular proliferative lesions and tubulointerstitial infiltration and decreased glomerular immune complex deposition in MRL/lpr mice. **a** Hematoxylin, eosin, and Periodic Acid-Schiff staining of kidney sections from wild-type (WT), MRL/lpr mice treated with vehicle, or MRL/lpr mice treated with an equivalent dose of TAC or HGC-TAC. **b** Immunofluorescence staining of kidney sections for C3, C1q, fibrinogen, IgG, IgA, and IgM. Original magnification ×200, Bar = 50 μm. **c** Scanning and transmission electron microscopy in each group. Red arrows indicate podocyte foot process effacements. Asterisk indicates mesangial deposition. Original magnification ×2000, ×8000 and ×10,000, respectively; Bar = 5 μm. This experiment was performed with 3 mice per group

Histologically, hematoxylin, eosin, and Periodic Acid-Schiff staining of kidney sections from MRL/lpr mice revealed conspicuous inflammatory interstitial infiltration, global proliferative lesions in the glomeruli, and thickened capillary walls, which indicated a diffuse/focal proliferative glomerular nephritis pathology (Fig. 3a). HGC-TAC treatment attenuated tubulointerstitial inflammation and glomerular injury compared to vehicle or TAC treatment alone. In vehicle- or TAC-treated lupus nephritis mice, mild mesangial and subendothelial immune depositions of complement factors (C3 and C1q, as well as fibrinogen, IgG, IgA, and IgM) were detected, whereas the kidneys of HGC-TAC treated MRL/lpr mice displayed decreased immune deposits (Fig. 3b). Additionally, HGC-TAC treatment in MRL/lpr mice attenuated podocyte foot process effacement, mesangial deposition, and widening compared to vehicle or TAC treatment alone (Fig. 3c). This explains the improvement of proteinuria in HGC-TAC-treated lupus mice. Our data suggested that HGC-TAC treatment improves renal histology and glomerular immune deposition in lupus nephritis.

### Treatment with HGC-TAC nanomicelles attenuated inflammation in lupus nephritis mice

**Fig. 4:**
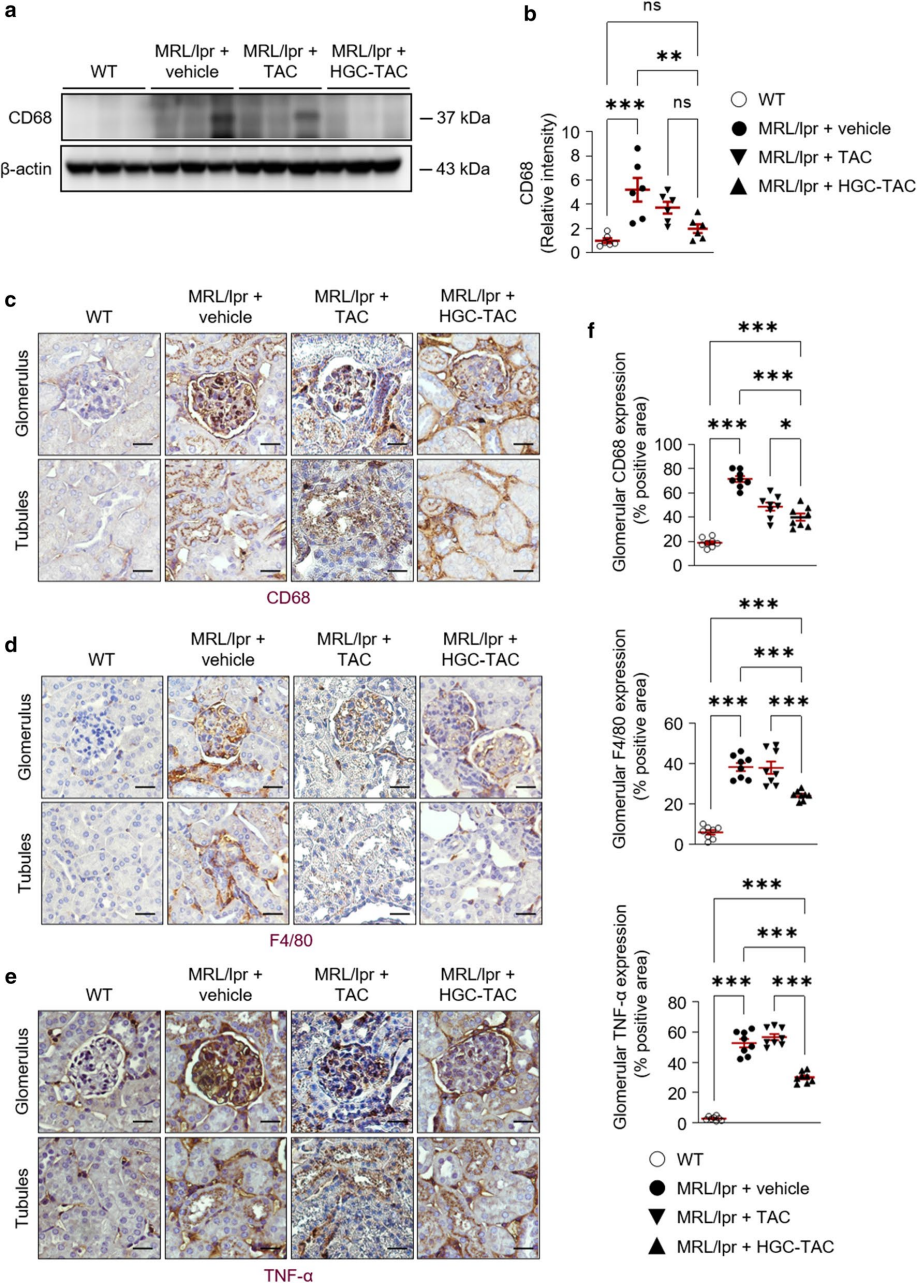
HGC-TAC diminished lupus-specific inflammation. **a** CD68 protein levels from wild-type (WT), MRL/lpr mice treated with vehicle, or MRL/lpr mice treated with an equivalent dose of TAC or HGC-TAC. **b** Relative protein intensities. The values for the WT vehicle-treated group are set to 1. (n = 6 mice/group). **c**–**e** Immunohistochemical staining of kidney sections for CD68, F4/80, and tumor necrosis factor-α (TNF-α) from each group. Magnification ×400, Bar = 25 μm. **f** Staining of CD68, F4/80, and TNF-α in the glomerulus was quantified and expressed as a percentage of positive glomerular area. Data are shown as mean ± SEM. *P < 0.05, **P < 0.01, and ***P < 0.001. *ns* not statistically significant

Next, we explored whether renal inflammatory protein and gene expression in lupus nephritis mice was modified by HGC-TAC treatment. HGC-TAC treatment in MRL/lpr mice tended to decrease the level of CD68^+^ cells, a marker for macrophages and monocytes, compared to vehicle or TAC treatment alone (Fig. 4a, b). Immunohistochemical staining showed that vehicle- or TAC-treated lupus nephritis kidneys had increased levels of CD68^+^ cells and F4/80+ mononuclear macrophages, whereas the levels of these cells were profoundly decreased in the glomerulus of HGC-TAC-treated lupus nephritis mice (Fig. 4c, d, and f).

**Fig. 5:**
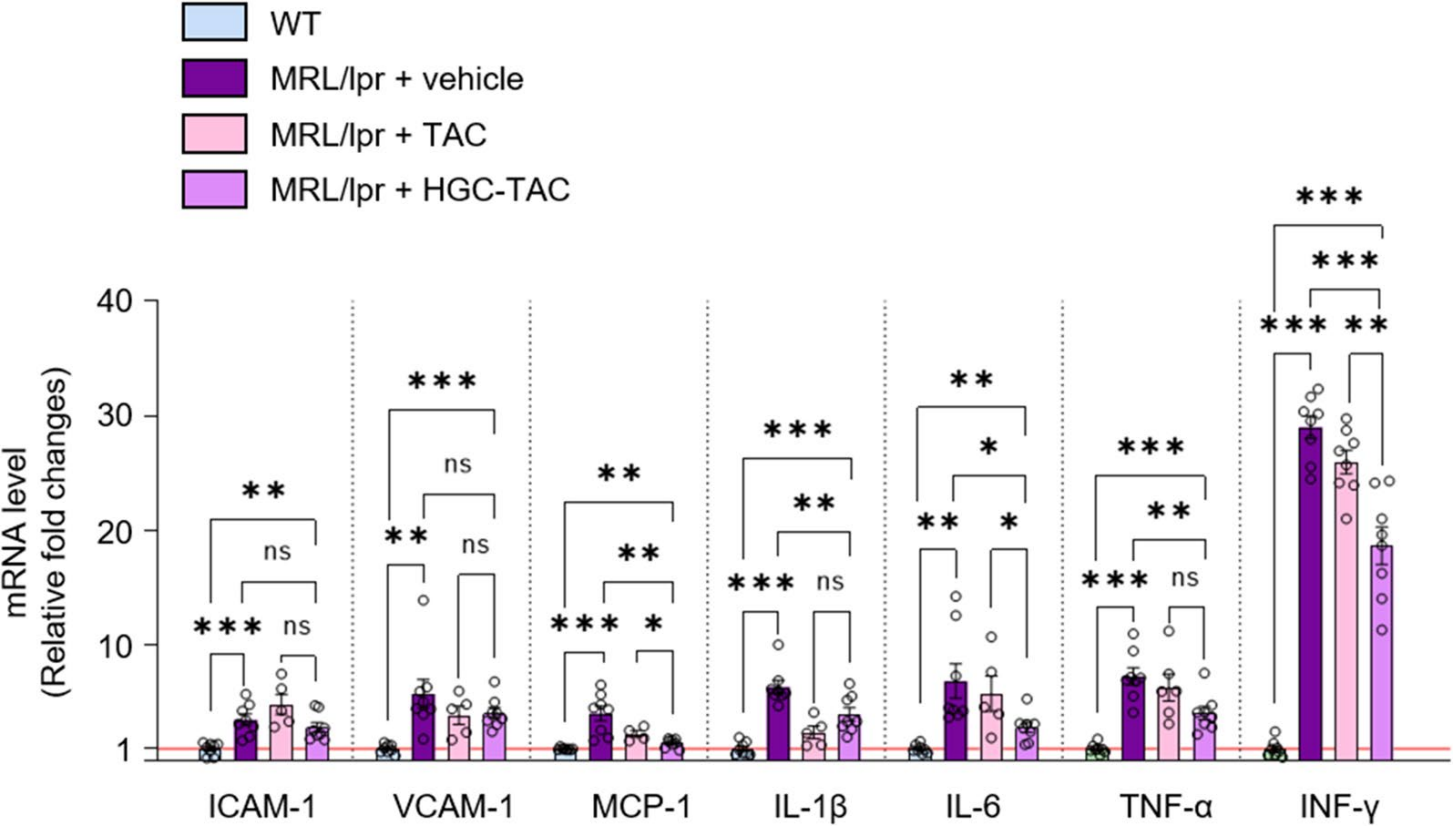
mRNA expression levels for inflammatory markers were lower in HGC-TAC-treated MRL/lpr mice. *ICAM-1* intercellular adhesion molecule-1, *VCAM-1* vascular cell adhesion molecule-1, *MCP-1* monocyte chemoattractant protein-1, *IL-1 β* interleukin 1β, *IL-6* interleukin 6, *TNF-α* tumor necrosis factor-α, *IMF-γ* interferon-γ (n = 6 mice/group). The values for WT vehicle-treated group are set to 1. Data are shown as means ± SEM. *P < 0.05, **P < 0.01, and ***P < 0.001. *ns* not statistically significant

Since interferon-γ (INF-γ) is a major pro-inflammatory T-cell cytokine that plays a pivotal role in the development of nephritis in MRL/lpr mice [28], we analyzed the mRNA expression of INF-γ using quantitative polymerase chain reaction (qPCR). The relative expression level of INF-γ was lower in the kidneys of HGC-TAC-treated MRL/lpr mice than that in the kidneys of vehicle-treated mice (Fig. 5). Consistent with these results, renal mRNA expression levels of inflammatory interleukin 1β (IL-1β), interleukin 6 (IL-6), monocyte chemoattractant protein-1 (MCP-1), and tumor necrosis factor-α (TNF-α) markers were lower in HGC-TAC-treated MRL/lpr mice than those observed in vehicle-treated lupus nephritis mice. Similarly, cell adhesion marker expression levels, such as intercellular adhesion molecule-1 (ICAM-1) and vascular cell adhesion molecule-1 (VCAM-1), were all low in the kidneys of HGC-TAC-treated lupus nephritis mice but did not reach statistical significance. In addition, HGC-TAC treatment significantly decreased the mRNA expression of MCP-1, IL-6, and IFN-γ compared to TAC treatment alone. Moreover, in line with the mRNA expression data, kidney section staining revealed increased TNF-α expression, especially in the glomerulus of vehicle- and TAC-treated MRL/lpr mice. Conversely, this expression was downregulated after HGC-TAC treatment in HGC-TAC-treated lupus mice (Fig. 4e, f). Overall, these data suggested that HGC-TAC treatment reduced glomerular inflammation in lupus nephritis mice.

### Treatment with HGC-TAC nanomicelles reduced renal glomerular fibrosis and apoptosis in lupus nephritis mice

**Fig. 6:**
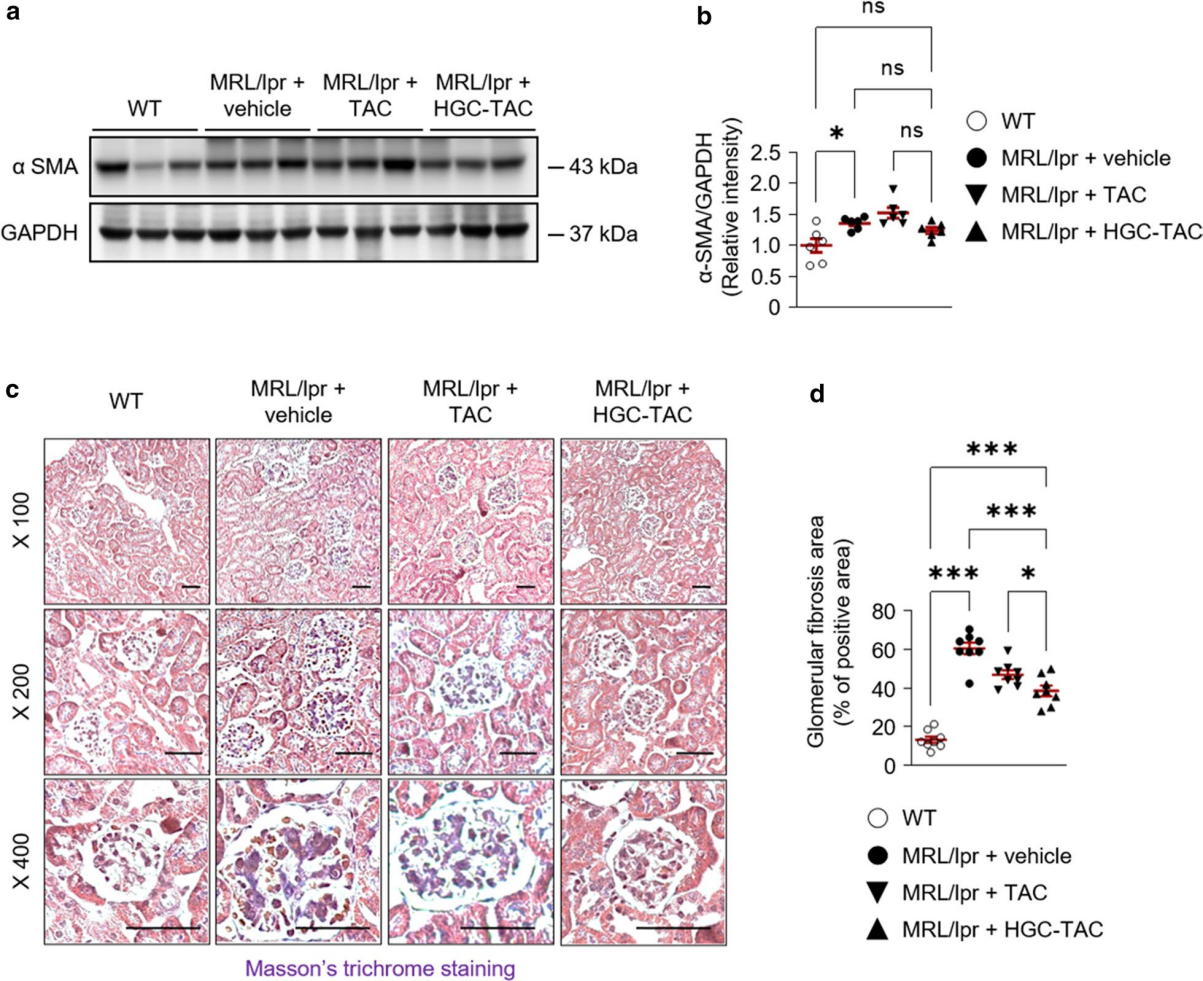
Treatment with HGC-TAC did improve renal glomerular fibrosis in MRL/lpr mice. **a** Western blot analysis of α smooth muscle actin (SMA) protein levels from wild-type (WT), MRL/lpr mice treated with vehicle, or MRL/lpr mice treated with an equivalent dose of TAC or HGC-TAC. **b** Relative protein intensities are presented. The values for the WT vehicle-treated group are set to 1. (n = 6 mice/group). **c** Masson’s trichrome staining of kidney sections from each group, Bar = 25 μm. **d** The fibrotic assessment of Masson’s trichrome-stained glomeruli was quantified and expressed as a percentage of positive glomerular area. All values are presented as mean ± SEM. *P < 0.05, **P < 0.01, and ***P < 0.001. *ns* not statistically significant

We next investigated whether HGC-TAC treatment affects renal fibrosis in lupus nephritis mice. α-smooth muscle actin levels were decreased in HGC-TAC-treated MRL/lpr mice than in vehicle- or TAC-treated MRL/lpr mice, but this effect was limited (Fig. 6a, b). However, kidney section staining with Masson’s trichrome stain showed differences in the extent of glomerular fibrosis between vehicle- or TAC-treated and HGC-TAC-treated lupus mice (Fig. 6c, d).

**Fig. 7:**
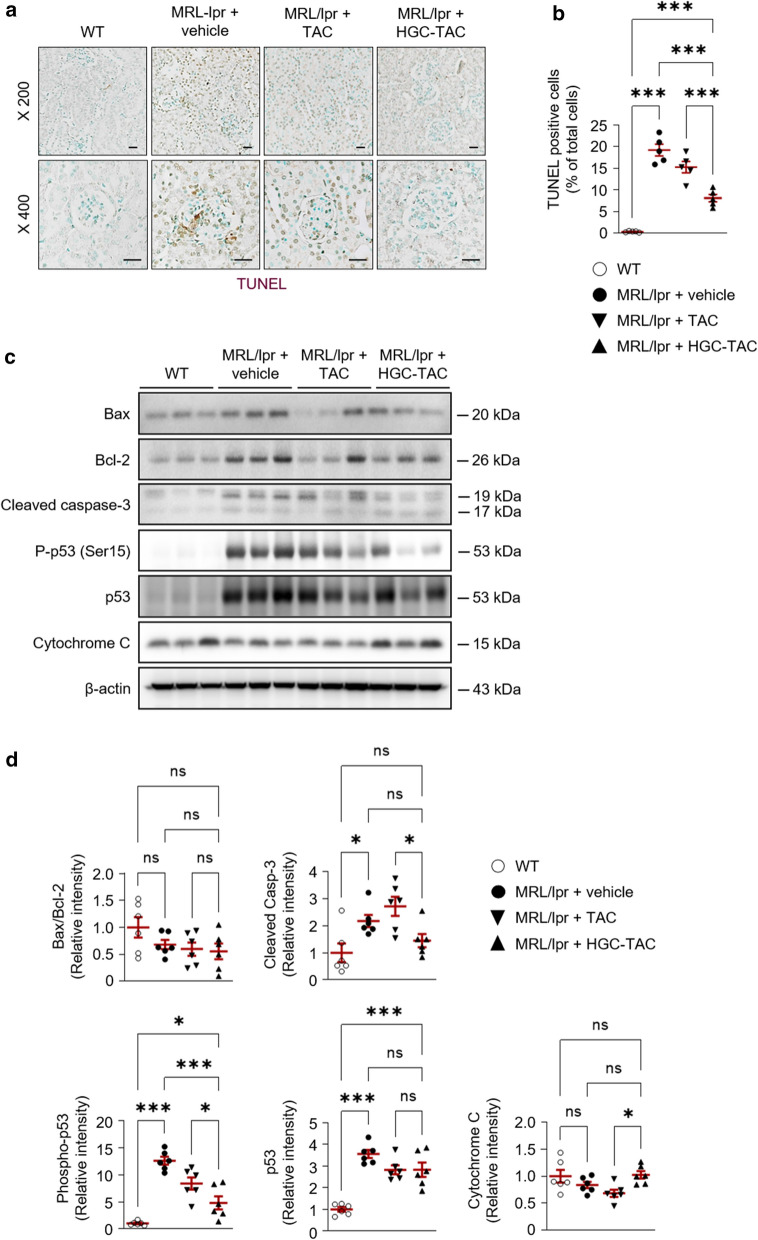
Treatment with HGC-TAC improved renal apoptosis in MRL/lpr mice. **a** TUNEL staining of kidney sections from wild-type (WT), MRL/lpr mice treated with vehicle, or MRL/lpr mice treated with an equivalent dose of TAC or HGC-TAC. Bar = 25 μm. **b** TUNEL positive cells were counted in each group and expressed as a percentage of total cells. **c** Western blot analysis of bax, bcl-2, cleaved caspase-3, P-p53 (Ser15), p53, and cytochrome *C* protein levels from each group. **d** Relative protein intensities are presented. The values for the WT vehicle-treated group are set to 1. (n = 6 mice/group). All values are presented as mean ± SEM. *P < 0.05, **P < 0.01, and ***P < 0.001. *ns* not statistically significant

To assess the effects of HGC-TAC treatment on renal apoptosis in lupus nephritis mice, we performed a terminal deoxynucleotidyl transferase dUTP nick end labeling (TUNEL) assay. TUNEL-positive cells were considerably increased in both the glomerulus and tubular epithelium of MRl/lpr mice. Notably, HGC-TAC treatment remarkably ameliorated TUNEL-positive cell counts in lupus nephritis mice (Fig. 7a, b). In contrast, HGC-TAC treatment did not affect the protein expression of the ratio of bax/bcl-2, cleaved caspase 3, and cytochrome compared to untreated lupus nephritis mice (Fig. 7c, d). However, the phosphorylation of p53 increased in vehicle- or TAC-treated MRL/lpr mice, whereas treatment with HGC-TAC significantly decreased the phosphorylation of p53. These findings suggest that HGC-TAC treatment may inhibit apoptosis in lupus nephritis mice via an intrinsic apoptotic pathway.

### HGC-TAC nanomicelle treatment regulates kidney protection in lupus nephritis mice via the TGF-β1/MAPK/NF-κB signaling pathway

**Fig. 8:**
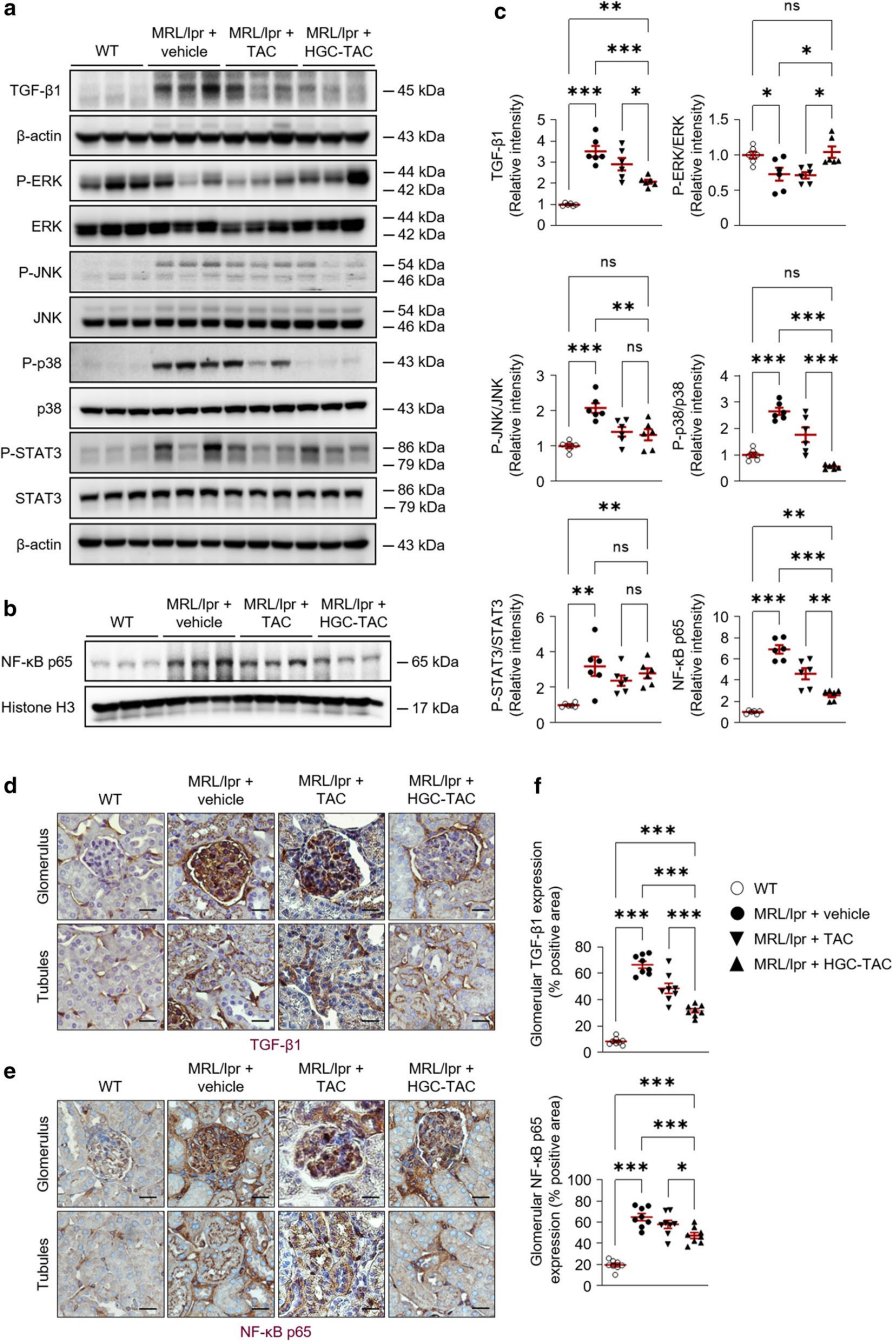
Treatment with HGC-TAC regulates the transforming growth factor (TGF)-β1/p38 mitogen-activated protein kinase (MAPK)/nuclear factor (NF)-κb signaling pathway in MRL/lpr mice. **a**, **b** Western blot analysis of TGF-β1, MAPKs, signal transducer, activator of transcription (STAT) 3, NF-κb, and protein levels from wild-type (WT), MRL/lpr mice treated with vehicle, or MRL/lpr mice treated with an equivalent dose of TAC or HGC-TAC. **c** Relative protein intensities are presented. The values for the WT vehicle-treated group are set to 1. (n = 6 mice/group). **d**, **e** Immunohistochemical staining of kidney sections for TGF-β1 and NF-κB p65 from each group. Original magnification ×400, Bar = 25 μm. **f** Staining for TGF-β1 and NF-κB p65 in the glomerulus was quantified and expressed as a percentage of positive glomerular area. All values are presented as mean ± SEM. *P < 0.05, **P < 0.01, and ***P < 0.001. *ns* not statistically significant

Since excessive activation of the transforming growth factor (TGF)-β1-mediated MAPK (mitogen-activated protein kinases) signaling pathway is involved in lupus nephritis development in both human and mouse [29, 30], we examined whether the HGC-TAC treatment-induced renoprotection observed in lupus nephritis mice is dependent on the TGF-β1/MAPK signaling pathway. HGC-TAC-treated mice showed a marked decrease in TGF-β1 levels compared to vehicle- or TAC-treated MRL/lpr mice (Fig. 8a). Compared to those in vehicle-treated wild-type mice, the phosphorylation levels of c-Jun NH2-terminal kinase (JNK) and p38 were higher in lupus nephritis mice; however, the phosphorylation of extracellular signal-regulated kinase (ERK) was lower. Among MAPKs, HGC-TAC treatment significantly reduced the phosphorylation of JNK and p38, while increased the phosphorylation of ERK (Fig. 8a, c). Although TAC-treated mice also showed similar effects, the increased expression of TGF-1β1 and p38 phosphorylation, and decreased ERK phosphorylation were significantly restored in HGC-TAC-treated mice. The phosphorylation of signal transducer and activator of transcription (STAT) 3 was also found to be upregulated in nephritic kidneys and is a critical component in the pathogenesis of lupus nephritis [31]. Although the expression of STAT3 phosphorylation was elevated in vehicle-treated MRL/lpr mice, it was unaffected by HGC-TAC treatment (Fig. 8a, c).

To further evaluate the downstream signaling pathway of TGF-β1/MAPK, we assessed nuclear factor-κB (NF-κB) signaling pathway alterations. Phosphorylation of p65 was increased in nephritic mice, whereas treatment with HGC-TAC resulted in a decrease in p65 phosphorylation (Fig. 8b, c). TAC treatment also decreased the p65 phosphorylation, but HGC-TAC-treated lupus mice exhibited lower expression than TAC-treated lupus mice. Consistent with these data, TGF-β1 and NF-κB staining were markedly increased in lupus nephritis glomerulus and tubules, whereas these expressions were attenuated in the HGC-TAC-treated lupus mice (Fig. 8d, e, and f). These data collectively indicate that HGC-TAC negatively regulates the TGF-β1/MAPK/NF-κB signaling pathway, providing kidney protection in lupus nephritis mice, but does not mediate the STAT3 signaling pathway.

## Discussion

In this study, we investigated the renoprotective effects and efficacy of HGC-TAC nanomicelle treatment in a MRl/lpr mouse model of lupus nephritis. Lupus-associated activity and proteinuria were attenuated by HGC-TAC treatment. In addition, HGC-TAC treatment mitigated renal dysfunction and histological injury, including glomerular proliferative lesions and tubulointerstitial infiltration. Furthermore, HGC-TAC administration reduced renal inflammation and accompanying inflammatory gene expression in the lupus nephritis mouse model. Additionally, HGC-TAC administration ameliorated increased glomerular fibrosis and renal apoptosis and appeared to regulate renal inflammation via the TGF-β1/MAPK/NF-κB signaling pathway. Regarding these renoprotective effects, HGC-TAC was more potent compared to an equivalent dose of TAC treatment alone.

Several studies have discussed the feasibility of site-specific drug delivery into the kidneys for the treatment of glomerulonephritis [24–26]. In ddY mice, a spontaneous animal model for IgA nephropathy, treatment with prednisolone phosphate-loaded liposomes showed better improvement in glomerular IgA and C3 depositions compared to ordinary prednisolone phosphate treatment characterized by the same dose and duration [24]. Similarly, dexamethasone loaded immunoliposomes were highly effective in improving renal function and decreasing glomerular crescent formation (without affecting blood glucose levels) in an anti-glomerular basement membrane glomerulonephritis model [26]. Moreover, a previous study demonstrated that a single intravenous injection of MMF containing immunoliposomes reduced mesangial cells in anti-Thy1.1 nephritis rats, a model of mesangial proliferative glomerulonephritis [25]. Therefore, targeted delivery of a steroid or MMF using immunoliposome may maintain the efficacy and quality of these drugs for kidney inflammation but minimize systemic side effects in the glomerulonephritis model.

However, kidney-targeted, nanomicelle-based TAC delivery methods for lupus nephritis models are yet to be developed. A recent study showed that monotherapy with TAC significantly diminished proteinuria and Toll-like receptor-7 expression and induced the suppression of IL-6 production in lupus nephritis mice [6]. Additionally, TAC monotherapy preserved renal function in nephritic mice by inhibiting podocyte apoptosis and stabilizing the actin cytoskeleton [7]. However, in these studies, lupus mice were given 0.1 to 1 mg/kg TAC daily by intragastric administration for 8 weeks [6, 7]. In the present study (compared to the results of long-term and frequent TAC administration via daily oral gavage), we showed that weekly intravenous injections of HGC-TAC (0.5 mg/kg TAC) nanomicelles alone inhibited renal inflammation and resulted in improved renal morphology and function in lupus nephritis mice. However, weekly treatment with an equivalent TAC dose without HGC nanomicelles exhibited suboptimal renoprotective effects in lupus nephritis mice compared to HGC-TAC treatment. Therefore, kidney-targeted HGC-TAC delivery can exert renoprotective effects using a smaller than conventional TAC dose with an extended administration interval. Since poor adherence to immunosuppressive therapy is common and is one of the most important factors limiting renal allograft survival following transplantation in lupus patients [32, 33], HGC-TAC nanocarriers may improve the drug adherence associated with reduced mortality [34]. Thus, our findings suggest HGC-TAC nanomicelles as a new therapeutic modality that can reduce pill burden or extend the interval of TAC administration in lupus nephritis patients.

Immunofluorescence staining of kidney biopsies showed substantial expression of TGF-β1 and increased urinary levels of TGF-β1, as reported in lupus patients [29, 35, 36]. Previous studies have shown that decreased ERK signaling is associated with the development of autoimmunity in lupus via the decrease in the activity of DNA methyltransferases and the consequent alteration of gene expression; of note, lupus-prone mice are characterized by the increased phosphorylation of JNK [37–39]. Cell survival and death may therefore be controlled by the opposing actions of the ERK and JNK pathways [40]. In addition, the activation of p38MAPK is involved in TGF‐β1‐mediated gene expression and apoptosis in MRL/lpr mice [30]. In line with the results of these studies, we found that the TGF-β1/JNK-p38MAPK signaling pathways were upregulated, while the expression of ERK was downregulated in vehicle-treated lupus mice. These altered signaling mediators were restored by treatment with HGC-TAC. Importantly, our results were also consistent with those of a previous study demonstrating that MAPK1 short interfering RNAs (with nanocarrier therapy) suppressed glomerular MAPK1 gene and protein expression in lupus nephritis mice [27]. NF-κB is associated with the onset of various inflammatory autoimmune diseases, including lupus nephritis [41]. The phosphorylation of NF-κB occurs in the cytoplasm, enhancing NF-κB transcriptional activity [42]. Thus, NF-κB signaling regulates the expression of numerous genes that play key roles in the inflammatory response during kidney injury [43]. Consequently, NF-κB signaling plays a pathological role in lupus nephritis, and interference with this signaling by HGC-TAC nanomicelle treatment contributes to renoprotective effects in lupus nephritis mice.

In conclusion, we have demonstrated that weekly treatment with HGC-TAC nanomicelles prevents kidney injury from advanced lupus nephritis by preventing inflammation, fibrosis, and apoptosis through the modulation of the TGF-β1/MAPK/NF-κB singling pathway. Although a key takeaway of this study is that a new therapeutic modality using a kidney-targeted, TAC-loaded nanocarrier may provide benefits for treating nephritis in lupus mice, the therapeutic efficacy of TAC-loaded nanocarriers remains limited to animal models. Further studies are needed to clarify the renoprotective effects of HGC-TAC nanomicelles in humans.

## Methods

### Synthesis of TAC-loaded HGC (HGC-TAC) nanomicelles

We synthesized glycol chitosan conjugated to 5β-cholanic acid micelles and loaded TAC, as described in our previous report [23]. Briefly, lyophilized glycol chitosan was conjugated to 5β-cholanic acid via EDC NHS chemistry. The retrieved sample (HGC) was lyophilized and stored for further use. TAC-loaded HGC (HGC-TAC) nanomicelles were prepared using a nanoprecipitation method. The prepared HGC was dissolved in distilled water. Under mild sonication, TAC prepared in methanol was added dropwise to the HGC solution. The drug-loaded sample (HGC-TAC) was dialyzed (MWCO: 12 to 14 KDa) against distilled water for 2 days, lyophilized, and stored until use. The amount of TAC in the HGC-TAC nanomicelles was measured using high-performance liquid chromatography (HPLC) (Shimadzu, Kyoto, Japan).

### Particle size, surface charge, stability, and FE-TEM analysis

The hydrodynamic particle size and surface charge of HGC-TAC were measured by dynamic light scattering (Zetasizer Nano series, Malvern Instruments, Malvern, UK). The colloidal stability of the nanomicelles in distilled water, PBS, and 10% fetal bovine serum were assessed using dynamic light scattering over 7 days. The size and morphology of nanomicelles were also measured using FE-TEM operated at 200 kV (JEM-2100 F, Tokyo, Japan).

### In vitro release of TAC

In vitro release of TAC from nanomicelles was studied in PBS and 10% FBS for 7 days. Samples were placed in a dialysis bag and kept in 20 ml release medium (PBS or 10% FBS) in a shaking incubator at 37 ℃. Samples were aliquoted at different time points and analyzed using HPLC.

### Determination of kidney and plasma TAC concentration

The concentration of TAC in the kidney and plasma was determined as previously described [23]. Briefly, 0.5 mg/kg of HGC-TAC was intravenously injected. Plasma was collected by retro-orbital sinus bleeding at different time points and centrifuged at 845×*g* for 10 min. An equal amount of 100% methanol was added to the plasma and centrifuged at 13,500×*g* for 2 min. The collected supernatant was used for HPLC analysis. To determine the concentration of TAC in the kidney, each kidney was homogenized in methanol using a TissueLyser II (Qiagen, Hilden, Germany). The homogenized solution was centrifuged at 13,500×*g* for 10 min, and the supernatant was then collected for HPLC analysis.

### In vivo biodistribution of nanomicelles

Female MRL/MpJ-*Fas*^*lpr*^ mice were intravenously injected with Flamma 675-conjugated HGC dissolved in PBS. The mice were euthanized, and their organs were collected at predetermined time points (1, 2, 3, 5, and 7 days) after a single intravenous injection. Fluorescence intensity was measured using a fluorescence-labeled organism bioimaging instrument (FOBI; NEO Science, Gyeonggi, Korea). The isolated kidneys were dehydrated with 20% sucrose in PBS for 4 h at 4 ℃ and embedded in an optimal cutting temperature compound. Frozen kidney sections of 20 μm were prepared. The sections were rehydrated with PBS at the time of immunostaining and were counterstained with 4′,6-diamidino-2-phenylindole. Images were acquired using a confocal microscope (LSM 800; Carl Zeiss, Oberkochen, Germany).

### Cellular uptake of nanomicelles

The human proximal tubular cells were seeded in Lab-Tek^®^ Chamber Slide and incubated overnight. The media was aspirated, and the cells were treated with HGC-F675 nanomicelles at different time points up to 6 h. Subsequently, the samples were removed, followed by washing with PBS and 4% paraformaldehyde fixation. The cells were stained with Hoechst 33342 and mounted with prolong gold antifade reagent. The fluorescence signal was visualized using confocal microscopy. Cells not treated with HGC-F675 were used as a negative control.

### Animal experiments

Mice were maintained in a 12-h light/dark cycle and had free access to standard chow (Damul Science, Daejeon, Korea) and tap water. Eight-week-old female MRL/MpJ and MRL/MpJ-*Fas*^*lpr*^ mice were purchased from the Jackson Laboratory (Bar Habor, ME, USA). Female MRL/MpJ-*Fas*^*lpr*^ mice were randomly assigned into three groups and given either vehicle, an equivalent dose of TAC, or HGC-TAC (0.5 mg/kg TAC) intravenously once per week for 8 weeks. Age- and sex-matched MRL/MpJ mice without *Fas*^*lpr*^ mutation were treated with vehicle and used as healthy controls (n = 6 mice per group) (Fig. 2a). Experiments were repeated at least twice.

### Measurement of serum anti-double-stranded DNA (anti-dsDNA) antibody, BUN, creatinine, and complement C3

The amount of IgG anti-dsDNA antibody in mouse sera was measured by ELISA using calf thymus dsDNA (5110; Alpha Diagnostic, San Antonio, TX). Urea, creatinine, and complement C3 were measured in serum using a Beckman Coulter AU5822 autoanalyzer (Beckman Coulter, Brea, CA) (Fig. 8).

### Measurement of urine protein and albumin-to-creatinine ratio

Urine samples were collected in metabolic cages to examine the levels of urinary protein and albumin excretion and ratios of urinary protein and albumin to creatinine. Urine creatinine was quantified using commercial kits from BioAssay Systems (Hayward, CA). Urine protein was assessed using Bradford’s method (DC Protein Assay, Bio-Rad Laboratories GmbH, Munich, Germany). Urine albumin was determined using a commercial assay from Bethyl Laboratory, Inc. (Montgomery, TX).

### Western blot analysis

Proteins extracted from mouse tissues were obtained by homogenization in ice-cold modified RIPA buffer (150 mM sodium chloride, 50 mM Tris–HCl (pH 7.4), 1 mM EDTA, 1% *v/v* Triton-X 100, 1% *w/v* sodium deoxycholic acid, 0.1% *v/v* SDS) and centrifuged at 4000×*g* for 15 min at 4 ℃. Western blot analysis was performed as described previously [44]. Densitometry was performed using Scion Image software (Scion Corporation, Frederick, MD). The primary and secondary antibodies used in western blotting are listed in Additional file 3: Table S1.

### Quantitative reverse transcription-polymerase chain reaction (qRT-PCR)

Total RNA was extracted using Trizol reagent (Invitrogen, Carlsbad, CA). cDNA was reverse transcribed from 5 μg of total RNA using SuperScript II Reverse Transcriptase as per the manufacturer’s instructions (Invitrogen). qRT-PCR analysis was performed using the SYBR green method [45]. The relative level of tissue mRNA was determined by qPCR using a Rotor-Gene Q (QIAGEN Sciences, Germantown, MD). The primers used in qRT-PCR are listed in Additional file 3: Table S2.

### Immunohistochemical and immunofluorescence staining

The kidneys were fixed in 4% paraformaldehyde, dehydrated using a graded series of ethanol, embedded in paraffin, sectioned (3 µm), and mounted on glass slides. Hematoxylin and eosin and Periodic Acid-Schiff staining were used to assess kidney histology. Periodic Acid-Schiff and Masson’s trichrome staining were performed according to the manufacturer’s instructions (Abcam, Cambridge, MA) [46]. For immunohistochemical staining, paraffin sections were dewaxed and rehydrated via a xylene/ethanol gradient followed by antigen retrieval (100 ℃ for 15 min in citrate buffer, pH 6.0) using Antigen Unmasking Solution (Vector Laboratories, Burlingame, CA). Sections were blocked with 2.5% bovine serum albumin in PBS and incubated with primary antibodies overnight at 4 °C and then with the appropriate horseradish peroxidase-conjugated secondary antibody. Sections were incubated in a 3,30-diaminobenzidine reaction solution (Abcam) and counterstained with hematoxylin. For immunofluorescence staining, frozen sections (5 μm) were fixed for 10 min in cold acetone and then stained with primary FITC-conjugated antibodies. Primary and secondary antibodies used for immunohistochemistry are listed in Additional file 3: Table S3. CD68, F4/80, TNF-α, TGF-β, and NF-κB p65-positive stains were quantified in 10 to 15 glomeruli in each section, and the positive glomerular areas were expressed as a percentage of the total area.

### Terminal deoxynucleotidyl transferase dUTP nick end labeling (TUNEL) assay

Apoptosis of tubular epithelial cells was detected via TUNEL staining using an ApopTag Plus Peroxidase In Situ Apoptosis Kit (S7110, Sigma-Aldrich, St. Louis, MO), according to the manufacturer’s instructions. TUNEL-positive cells were quantified in each section, and the number of positive cells was expressed as a percentage of the total cells.

### Transmission electron microscopy

Small kidney cortices were fixed in 4% glutaraldehyde and 1% paraformaldehyde, dehydrated, and embedded in Spurr resin. Glomeruli were localized in semi-thin sections stained with toluidine blue. Ultrathin sections, with one or two glomeruli per tissue specimen, were stained with lead citrate for transmission electron microscopy. Four to ten photographs covering one or two glomerular cross-sections were captured using a JEM-1400 transmission electron microscope (JEOL, Peabody, MA). The images obtained had a final magnification of approximately ×10,000.

### Scanning electron microscopy

Small cubes of kidney cortex fixed in 2.5% glutaraldehyde were immersed in 1% osmium tetroxide in phosphate buffer for 2 h. Following dehydration with a graded series of ethanol, specimens were transferred into hexamethyldisilazane for chemical drying. After mounting on aluminum stubs with carbon paste, the dried specimens were coated with gold using an ion sputter coater (SPT-20, COXEM, Daejeon, Korea) and observed with an EM-30AX scanning electron microscope (COXEM, Daejeon, Korea).

### Statistical analyses

The results are expressed as mean ± standard error of the mean. The statistical significance of differences was determined using unpaired Student’s t-test or one-way analysis of variance followed by post hoc Tukey’s (honestly significant difference, or HSD) test. All statistical analyses were performed using GraphPad Prism 9 (GraphPad Software, San Diego, CA).

## Supplementary Information

**Additional file 1: Fig. S1** Characterization of HGC-TAC nanomicelles. (a, b) The distribution of hydrodynamic diameter and zeta potential of HGC-TAC nanomicelles. (c) The colloidal stability (hydrodynamic diameter, zeta potential, and polydispersity index) of HGC-TAC nanomicelles in distilled water, phosphate-buffered saline (PBS), and 10% fetal bovine saline (FBS) was assessed by time-dependent changes. Data are shown as mean ± SEM. (d) The cellular uptake of HGC-F675 nanomicelles in human proximal tubular cells at different time points. Bar = 20 μm. Note that negative control was cells not treated with HGC-F675.

**Additional file 2: Fig. S2** In vitro and in vivo release profiles of TAC from HGC-TAC nanomicelles. (a, b) In vitro cumulative release of TAC from HGC-TAC, performed in PBS (pH 7.4) and 10% FBS via the dialysis bag diffusion method. (c) TAC concentration in plasma and kidney tissues after a single intravenous injection of HGC-TAC nanomicelles in MRL/lpr mice (n = 3 mice/group). All values are presented as mean ± SEM**.**

**Additional file 3: Table S1.** List of primary and secondary antibodies for western blotting. **Table S2.** List of primers sequences used for qRT-PCR. **Table S3.** List of primary and secondary antibodies for immunohistochemistry.

## Data Availability

The data are available in the main manuscript, additional information files, and from the corresponding authors upon reasonable request.

## Abbreviations

SLE: Systemic lupus erythematosus
TAC: Tacrolimus
MMF: Mycophenolate mofetil
HGC: Hydrophobically modified glycol chitosan
HGC-TAC: Nanomicelles loaded with tacrolimus
FE-TEM: Field-emission transmission electron microscopy
HGC-F675: Flamma 675-conjugated HGC
PBS: Phosphate-buffered saline
FBS: Fetal bovine serum
qPCR: Quantitative polymerase chain reaction
INF-γ: Interferon-γ
IL-1β: Interleukin 1β
IL-5: Interleukin 6
MCP-1: Monocyte chemoattractant protein-1
TNF-α: Tumor necrosis factor-α
ICAM-1: Intercellular adhesion molecule-1
VCAM-1: Vascular cell adhesion molecule-1
TUNEL: Terminal deoxynucleotidyl transferase dUTP nick end labeling
TGF: Transforming growth factor
MAPK: Mitogen-activated protein kinases
JNK: C-Jun NH2-terminal kinase
ERK: Extracellular signal-regulated kinase
STAT: Signal transducer and activator of transcription
HPLC: High-performance liquid chromatography
NK-κB: Nuclear factor-κB
