# Supplementary figures and images for: Glycol chitosan-based tacrolimus-loaded nanomicelle therapy ameliorates lupus nephritis
Source: J Nanobiotechnology. 2021 Apr 17;19:109. doi: 10.1186/s12951-021-00857-w (PMC8052756; doi:10.1186/s12951-021-00857-w)

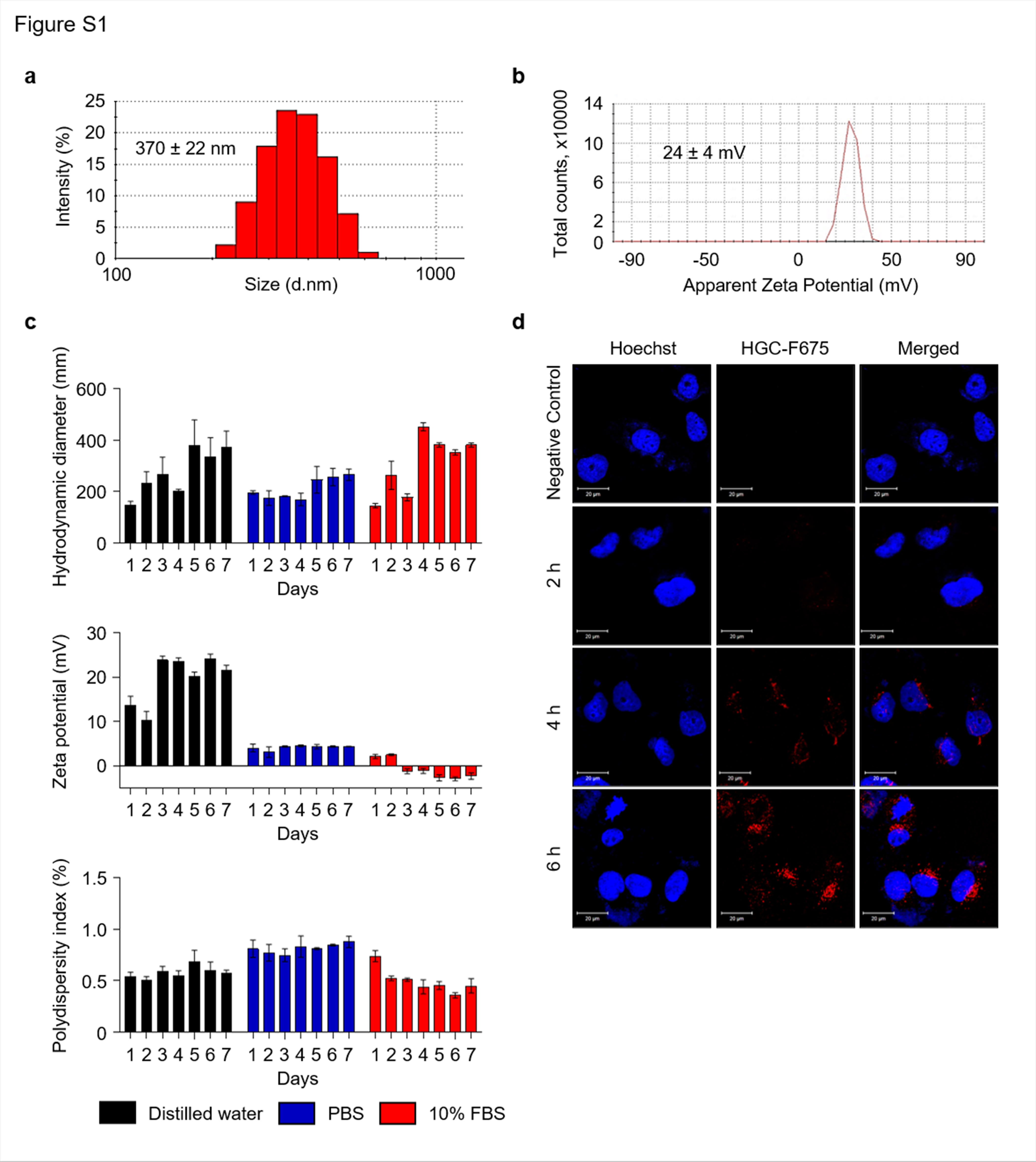

Supplement: Supplementary file 1 — Additional file 1: Fig. S1 Characterization of HGC-TAC nanomicelles. (a, b) The distribution of hydrodynamic diameter and zeta potential of HGC-TAC nanomicelles. (c) The colloidal stability (hydrodynamic diameter, zeta potential, and polydispersity index) of HGC-TAC nanomicelles in distilled water, phosphate-buffered saline (PBS), and 10% fetal bovine saline (FBS) was assessed by time-dependent changes. Data are shown as mean ± SEM. (d) The cellular uptake of HGC-F675 nanomicelles in human proximal tubular cells at different time points. Bar = 20 μm. Note that negative control was cells not treated with HGC-F675. [file 12951_2021_857_MOESM1_ESM.tif]

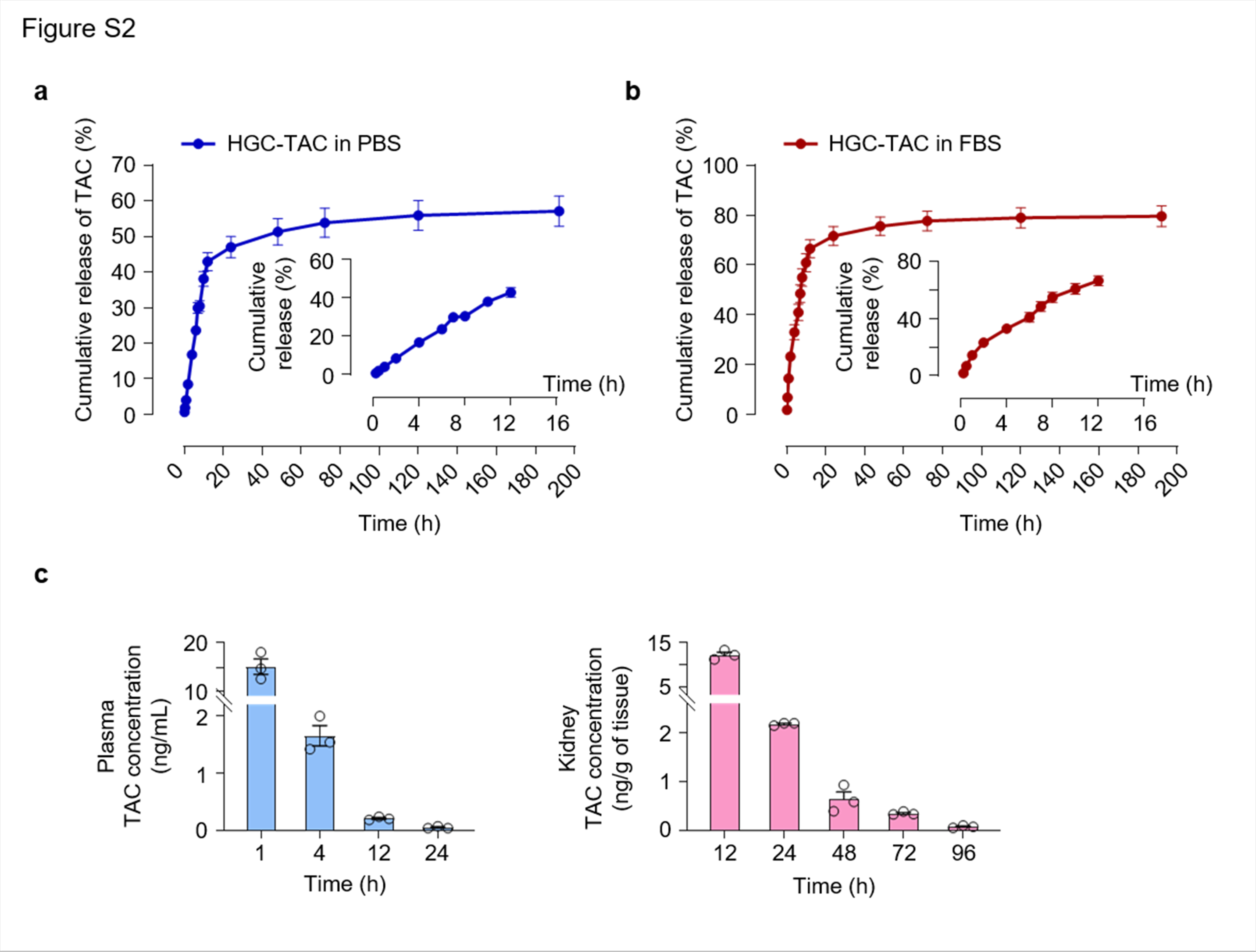

Supplement: Supplementary file 2 — Additional file 2: Fig. S2 In vitro and in vivo release profiles of TAC from HGC-TAC nanomicelles. (a, b) In vitro cumulative release of TAC from HGC-TAC, performed in PBS (pH 7.4) and 10% FBS via the dialysis bag diffusion method. (c) TAC concentration in plasma and kidney tissues after a single intravenous injection of HGC-TAC nanomicelles in MRL/lpr mice (n = 3 mice/group). All values are presented as mean ± SEM. [file 12951_2021_857_MOESM2_ESM.tif]
