# Supplementary material for: Glycol chitosan-based tacrolimus-loaded nanomicelle therapy ameliorates lupus nephritis
Source: J Nanobiotechnology. 2021 Apr 17;19:109. doi: 10.1186/s12951-021-00857-w (PMC8052756; doi:10.1186/s12951-021-00857-w)
Supplement: Supplementary file 3 — Additional file 3: Table S1. List of primary and secondary antibodies for western blotting. Table S2. List of primers sequences used for qRT-PCR. Table S3. List of primary and secondary antibodies for immunohistochemistry. [file 12951_2021_857_MOESM3_ESM.docx]

**Supplementary Table S1**. List of primary and secondary antibodies for western blotting.

| Name | Host species | Reactivity | Cat. No. | Manufacture |
| --- | --- | --- | --- | --- |
| Transforming growth factor-β1 | Rabbit | Human, Mouse, Rabbit | #3711 | CST |
| BAX | Rabbit | H,M,R,MK | #2772 | CST |
| Bcl-2 | Rabbit | H,M | #3498 | CST |
| P53 | Rabbit | H,Mk | #9282 | CST |
| Phospho-p53 | Rabbit | H,M,R,Mk | #9284 | CST |
| Cleaved-caspase 3 | Rabbit | H,M,R,Mk | #9661 | CST |
| Cytochrome C | Rabbit | H,M,R,Dm | Sc-7159 | Santa Cruz |
| α-Smooth muscle actin | Mouse | R, Gp, M, C, H, R | A2547 | Sigma-Aldrich |
| CD68 | Mouse | M, R, H | #31630 | Abcam |
| Phospho ERK1/2 | Rabbit | H, M, R, Hm, C, Z | 9101s | CST |
| ERK1/2 | Rabbit | H, M, R, Hm, Z | #9102 | CST |
| Phospho P38 | Rabbit | H, M, R, Mk, Dm, Pg, Sc | #9215 | CST |
| P38 | Rabbit | H, M, R, Mk, Gp | #9212 | CST |
| Phospho JNK1/2 | Rabbit | H, M, R, Mk, Hm | #9251s | CST |
| JNK1/2 | Rabbit | H, M, R, Hm, Z | #9252 | CST |
| Phospho STAT3 | Rabbit | H, M, R, C | #9131 | CST |
| STAT3 | Rabbit | H, M, R | #9132 | CST |
| Nuclear factor-κB p65 | Rabbit | H,M,R,MK | #8242 | CST |
| Nephrin | Mouse | H,M,R | Sc-376522 | Santa Cruz |
| β-actin | Rabbit | Human, Mouse | 4967 | CST |
| GAPDH | Mouse | H, M, R, Hm, C | G9295 | Sigma-Aldrich |
| Goat IgG, HRP-linked |  | Goat IgG | AP106P | Millipore |
| Rabbit IgG, HRP-linked |  | Rabbit IgG | 7074s | CST |
| Mouse IgG, HRP-linked |  | Mouse IgG | 7076s | CST |

ERK, extracellular signal-regulated kinase; JNK, c-Jun NH2-terminal kinase; STAT, signal transducer and activator of transcription; GAPDH, Glyceraldehyde 3-phosphate dehydrogenase

**Supplementary Table S2**. List of primers sequences used for qRT-PCR

| Gene | Species | Forward | Reverse |
| --- | --- | --- | --- |
| *Icam1* | Mouse | AACTTTTCAGCTCCGGTCCTG | TCAGTGTGAATTGGACCTGCG |
| *Vcam1* | Mouse | TCTCTCAGGAAATGCCACCC | CACAGCCAATAGCAGCACAC |
| *Mcp1* | Mouse | ATCCCAATGAGTAGGCTGGAGAGC | CAGAAGTGCTTGAGGTGGTTGTG |
| *Il-1β* | Mouse | AACCTGCTGGTGTGTGACGTTC | CAGCACGAGGCTTTTTTGTTGT |
| *Il-6* | Mouse | ACAACCACGGCCTTCCCTACTT | CACGATTTCCCAGAGAACATGTG |
| *Tnfα* | Mouse | GCATGATCCGCGACGTGGAA | AGATCCATGCCGTTGGCCAG |
| *Tgfb1* | Mouse | CAACAATTCCTGGCGTTACCTTGG | GAAAGCCCTGTATTCCGTCTCCTT |
| *Gapdh* | Mouse | TGTGTCCGTCGTGGATCTGA | GATGCCTGCTTCACCACCTT |

qRT-PCR, Quantitative reverse transcription-polymerase chain reaction

**Supplementary Table S3**. List of primary and secondary antibodies for immunohistochemistry.

| Name | Host species | Reactivity | Titer | Cat. No. | Manufacture |
| --- | --- | --- | --- | --- | --- |
| Transforming growth factor-β1 | Rabbit | H, M | 1:800 | #3711 | CST |
| Tumor necrosis factor α | Rabbit | H | 1:400 | 6945s | CST |
| Nuclear factor κB p65 | Rabbit | H, M, R, Mk | 1:800 | #8242 | CST |
| F4/80 | Rat | Mouse |  | MCA497GA | Bio-Rad |
| Goat IgG, HRP-linked |  | Goat IgG |  | AP106P | Millipore |
| Rabbit IgG, HRP-linked |  | Rabbit IgG |  | K5007 | Dako |
| Mouse IgG, HRP-linked |  | Mouse IgG |  | K5007 | Dako |

TUNEL, terminal deoxynucleotidyl transferase dUTP nick end labeling
